# Supplementary material for: Genome-wide screens identify SEL1L as an intracellular rheostat controlling collagen turnover
Source: Nat Commun. 2024 Feb 20;15:1531. doi: 10.1038/s41467-024-45817-8 (PMC10879544; doi:10.1038/s41467-024-45817-8)
Supplement: Supplementary file 1 — Supplementary Information [file 41467_2024_45817_MOESM1_ESM.pdf]

Supplementary Information

Genome-wide screens identify SEL1L as an intracellular rheostat controlling collagen turnover

Michael J. Podolsky[1,*], Benjamin Kheyfets[1], Monika Pandey[1], Afaq H. Beigh[1], Christopher D. Yang[2], Carlos O. Lizama[2], Ritwik Datta[2], Liangguang L. Lin[3], Zhihong Wang[3], Paul J. Wolters[4], Michael T. McManus[5], Ling Qi[3], Kamran Atabai[2,4,6,*]

*Co-corresponding author

Corresponding author list:

Michael J. Podolsky, email: mip9227@med.cornell.edu

Kamran Atabai, email: kamran.atabai@ucsf.edu

Affiliations

1- Department of Medicine, Weill Cornell Medical College, New York, NY, USA.

2- Cardiovascular Research Institute, University of California, San Francisco, CA, USA.

3- Department of Molecular Physiology and Biological Physics, University of Virginia School of Medicine, Charlottesville, VA, USA.

4- Department of Medicine, University of California, San Francisco, CA, USA.

5- Department of Microbiology and Immunology and UCSF Diabetes Center, University of California, San Francisco, CA, USA.

6- Lung Biology Center, University of California, San Francisco, CA, USA.

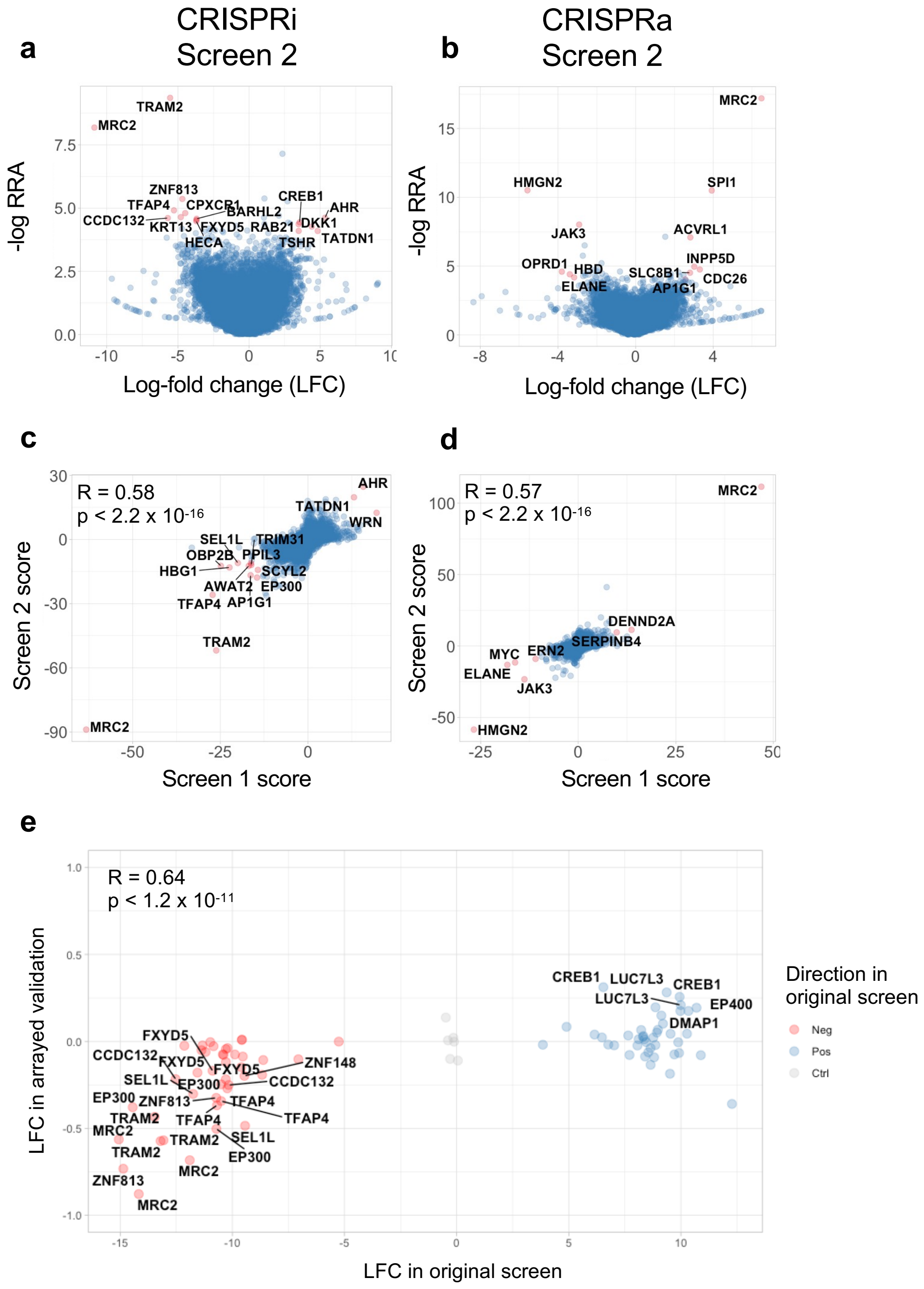

a
CRISPRi
Screen 2
b
CRISPRa
Screen 2
-log RRA
Log-fold change (LFC)
TRAM2
MRC2
ZNF813
TFAP4
CPXCR1
BARHL2
CCDC132
KRT13
FXYD5
RAB21
HECA
CREB1
AHR
DKK1
TSHR
TATDN1
HMGN2
SPI1
JAK3
ACVRL1
OPRD1
HBD
INPP5D
SLC8B1
CDC26
ELANE
AP1G1
c
d
Screen 2 score
Screen 1 score
R = 0.58
p < 2.2 x 10-16
SEL1L
TRIM31
OBP2B
PPIL3
HBG1
SCYL2
AWAT2
EP300
TFAP4
AP1G1
WRN
R = 0.57
DENND2A
SERPINB4
MYC
ERN2
e
R = 0.64
p < 1.2 x 10-11
LFC in arrayed validation
LFC in original screen
CREB1
LUC7L3
EP400
DMAP1
ZNF148
SEL1L
EP300
Direction in
original screen
Neg
Pos
Ctrl

Supplementary Figure 1. Re-screens are correlated to original CRISPR screen results. (a) Volcano plot of CRISPRi re-screen (Screen 2). (b) Volcano plot of CRISPRa re-screen (Screen 2). (c) Comparison of gene-level overall phenotype scores between CRISPRi screens 1 and 2. (d) Comparison of gene-level overall phenotype scores between CRISPRa screens 1 and 2. (e) Comparison of arrayed log-fold change in arrayed validation vs. original pooled screen log-fold change; each point represents unique guide sequence. 65/84 guides demonstrated concordance with original pooled screen data (77%). Pearson correlation coefficient is shown. Source data are provided in Supp. Data 1 and as a Source Data file.

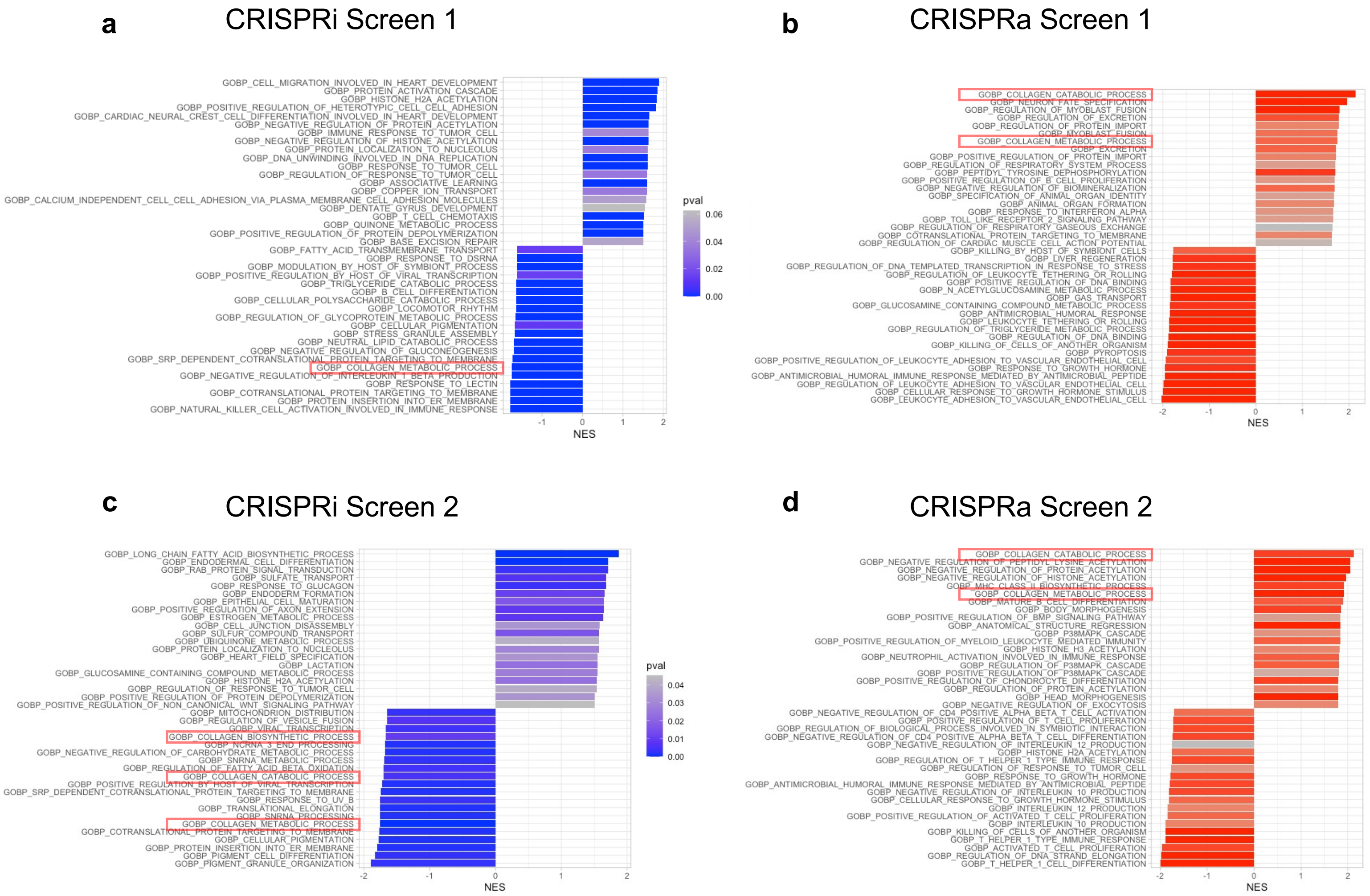
a
CRISPRi Screen 1
GOBP_CELL_MIGRATION_INVOLVED_IN_HEART_DEVELOPMENT
GOBP_PROTEIN_ACTIVATION_CASCADE
GOBP_HISTONE_H2A_ACETYLATION
GOBP_POSITIVE_REGULATION_OF_HETEROTYPIC_CELL_CELL_ADHESION
GOBP_CARDIAC_NEURAL_CREST_CELL_DIFFERENTIATION_INVOLVED_IN_HEART_DEVELOPMENT
GOBP_NEGATIVE_REGULATION_OF_PROTEIN_ACETYLATION
GOBP_IMMUNE_RESPONSE_TO_TUMOR_CELL
GOBP_NEGATIVE_REGULATION_OF_HISTONE_ACETYLATION
GOBP_PROTEIN_LOCALIZATION_TO_NUCLEOLUS
GOBP_DNA_UNWINDING_INVOLVED_IN_DNA_REPLICATION
GOBP_RESPONSE_TO_TUMOR_CELL
GOBP_REGULATION_OF_RESPONSE_TO_TUMOR_CELL
GOBP_ASSOCIATIVE_LEARNING
GOBP_COPPER_ION_TRANSPORT
GOBP_CALCIUM_INDEPENDENT_CELL_CELL_ADHESION_VIA_PLASMA_MEMBRANE_CELL_ADHESION_MOLECULES
GOBP_DENTATE_GYRUS_DEVELOPMENT
GOBP_T_CELL_CHEMOTAXIS
GOBP_QUINONE_METABOLIC_PROCESS
GOBP_POSITIVE_REGULATION_OF_PROTEIN_DEPOLYMERIZATION
GOBP_BASE_EXCISION_REPAIR
GOBP_FATTY_ACID_TRANSMEMBRANE_TRANSPORT
GOBP_RESPONSE_TO_DSRNA
GOBP_MODULATION_BY_HOST_OF_SYMBIONT_PROCESS
GOBP_POSITIVE_REGULATION_BY_HOST_OF_VIRAL_TRANSCRIPTION
GOBP_TRIGLYCERIDE_CATABOLIC_PROCESS
GOBP_B_CELL_DIFFERENTIATION
GOBP_CELLULAR_POLYSACCHARIDE_CATABOLIC_PROCESS
GOBP_LOCOMOTOR_RHYTHM
GOBP_REGULATION_OF_GLYCOPROTEIN_METABOLIC_PROCESS
GOBP_CELLULAR_PIGMENTATION
GOBP_STRESS_GRANULE_ASSEMBLY
GOBP_NEUTRAL_LIPID_CATABOLIC_PROCESS
GOBP_NEGATIVE_REGULATION_OF_GLUCONEOGENESIS
GOBP_SRP_DEPENDENT_COTRANSLATIONAL_PROTEIN_TARGETING_TO_MEMBRANE
GOBP_COLLAGEN_METABOLIC_PROCESS
GOBP_NEGATIVE_REGULATION_OF_INTERLEUKIN_1_BETA_PRODUCTION
GOBP_RESPONSE_TO_LECTIN
GOBP_COTRANSLATIONAL_PROTEIN_TARGETING_TO_MEMBRANE
GOBP_PROTEIN_INSERTION_INTO_ER_MEMBRANE
GOBP_NATURAL_KILLER_CELL_ACTIVATION_INVOLVED_IN_IMMUNE_RESPONSE
-1
0
1
2
NES
pval
0.06
0.04
0.02
0.00
b
CRISPRa Screen 1
GOBP_COLLAGEN_CATABOLIC_PROCESS
GOBP_NEURON_FATE_SPECIFICATION
GOBP_REGULATION_OF_MYOBLAST_FUSION
GOBP_REGULATION_OF_EXCRETION
GOBP_REGULATION_OF_PROTEIN_IMPORT
GOBP_MYOBLAST_FUSION
GOBP_COLLAGEN_METABOLIC_PROCESS
GOBP_EXCRETION
GOBP_POSITIVE_REGULATION_OF_PROTEIN_IMPORT
GOBP_REGULATION_OF_RESPIRATORY_SYSTEM_PROCESS
GOBP_PEPTIDYL_TYROSINE_DEPHOSPHORYLATION
GOBP_POSITIVE_REGULATION_OF_B_CELL_PROLIFERATION
GOBP_NEGATIVE_REGULATION_OF_BIOMINERALIZATION
GOBP_SPECIFICATION_OF_ANIMAL_ORGAN_IDENTITY
GOBP_ANIMAL_ORGAN_FORMATION
GOBP_RESPONSE_TO_INTERFERON_ALPHA
GOBP_TOLL_LIKE_RECEPTOR_2_SIGNALING_PATHWAY
GOBP_REGULATION_OF_RESPIRATORY_GASEOUS_EXCHANGE
GOBP_COTRANSLATIONAL_PROTEIN_TARGETING_TO_MEMBRANE
GOBP_REGULATION_OF_CARDIAC_MUSCLE_CELL_ACTION_POTENTIAL
GOBP_KILLING_BY_HOST_OF_SYMBIONT_CELLS
GOBP_LIVER_REGENERATION
GOBP_REGULATION_OF_DNA_TEMPLATED_TRANSCRIPTION_IN_RESPONSE_TO_STRESS
GOBP_REGULATION_OF_LEUKOCYTE_TETHERING_OR_ROLLING
GOBP_POSITIVE_REGULATION_OF_DNA_BINDING
GOBP_N_ACETYLGLUCOSAMINE_METABOLIC_PROCESS
GOBP_GAS_TRANSPORT
GOBP_GLUCOSAMINE_CONTAINING_COMPOUND_METABOLIC_PROCESS
GOBP_ANTIMICROBIAL_HUMORAL_RESPONSE
GOBP_LEUKOCYTE_TETHERING_OR_ROLLING
GOBP_REGULATION_OF_TRIGLYCERIDE_METABOLIC_PROCESS
GOBP_REGULATION_OF_DNA_BINDING
GOBP_KILLING_OF_CELLS_OF_ANOTHER_ORGANISM
GOBP_PYROPTOSIS
GOBP_POSITIVE_REGULATION_OF_LEUKOCYTE_ADHESION_TO_VASCULAR_ENDOTHELIAL_CELL
GOBP_RESPONSE_TO_GROWTH_HORMONE
GOBP_ANTIMICROBIAL_HUMORAL_IMMUNE_RESPONSE_MEDIATED_BY_ANTIMICROBIAL_PEPTIDE
GOBP_REGULATION_OF_LEUKOCYTE_ADHESION_TO_VASCULAR_ENDOTHELIAL_CELL
GOBP_CELLULAR_RESPONSE_TO_GROWTH_HORMONE_STIMULUS
GOBP_LEUKOCYTE_ADHESION_TO_VASCULAR_ENDOTHELIAL_CELL
-2
-1
0
1
2
NES
pval
0.015
0.010
0.005
0.000
c
CRISPRi Screen 2
GOBP_LONG_CHAIN_FATTY_ACID_BIOSYNTHETIC_PROCESS
GOBP_ENDODERMAL_CELL_DIFFERENTIATION
GOBP_RAB_PROTEIN_SIGNAL_TRANSDUCTION
GOBP_SULFATE_TRANSPORT
GOBP_RESPONSE_TO_GLUCAGON
GOBP_ENDODERM_FORMATION
GOBP_EPITHELIAL_CELL_MATURATION
GOBP_POSITIVE_REGULATION_OF_AXON_EXTENSION
GOBP_ESTROGEN_METABOLIC_PROCESS
GOBP_CELL_JUNCTION_DISASSEMBLY
GOBP_SULFUR_COMPOUND_TRANSPORT
GOBP_UBIQUINONE_METABOLIC_PROCESS
GOBP_PROTEIN_LOCALIZATION_TO_NUCLEOLUS
GOBP_HEART_FIELD_SPECIFICATION
GOBP_LACTATION
GOBP_GLUCOSAMINE_CONTAINING_COMPOUND_METABOLIC_PROCESS
GOBP_HISTONE_H2A_ACETYLATION
GOBP_REGULATION_OF_RESPONSE_TO_TUMOR_CELL
GOBP_POSITIVE_REGULATION_OF_PROTEIN_DEPOLYMERIZATION
GOBP_POSITIVE_REGULATION_OF_NON_CANONICAL_WNT_SIGNALING_PATHWAY
GOBP_MITOCHONDRION_DISTRIBUTION
GOBP_REGULATION_OF_VESICLE_FUSION
GOBP_VIRAL_TRANSCRIPTION
GOBP_COLLAGEN_BIOSYNTHETIC_PROCESS
GOBP_NCRNA_3_END_PROCESSING
GOBP_NEGATIVE_REGULATION_OF_CARBOHYDRATE_METABOLIC_PROCESS
GOBP_SNRNA_METABOLIC_PROCESS
GOBP_REGULATION_OF_FATTY_ACID_BETA_OXIDATION
GOBP_COLLAGEN_CATABOLIC_PROCESS
GOBP_POSITIVE_REGULATION_BY_HOST_OF_VIRAL_TRANSCRIPTION
GOBP_SRP_DEPENDENT_COTRANSLATIONAL_PROTEIN_TARGETING_TO_MEMBRANE
GOBP_RESPONSE_TO_UV_B
GOBP_TRANSLATIONAL_ELONGATION
GOBP_SNRNA_PROCESSING
GOBP_COLLAGEN_METABOLIC_PROCESS
GOBP_COTRANSLATIONAL_PROTEIN_TARGETING_TO_MEMBRANE
GOBP_CELLULAR_PIGMENTATION
GOBP_PROTEIN_INSERTION_INTO_ER_MEMBRANE
GOBP_PIGMENT_CELL_DIFFERENTIATION
GOBP_PIGMENT_GRANULE_ORGANIZATION
-2
-1
0
1
2
NES
pval
0.04
0.03
0.02
0.01
0.00
d
CRISPRa Screen 2
GOBP_COLLAGEN_CATABOLIC_PROCESS
GOBP_NEGATIVE_REGULATION_OF_PEPTIDYL_LYSINE_ACETYLATION
GOBP_NEGATIVE_REGULATION_OF_PROTEIN_ACETYLATION
GOBP_NEGATIVE_REGULATION_OF_HISTONE_ACETYLATION
GOBP_MHC_CLASS_II_BIOSYNTHETIC_PROCESS
GOBP_COLLAGEN_METABOLIC_PROCESS
GOBP_MATURE_B_CELL_DIFFERENTIATION
GOBP_BODY_MORPHOGENESIS
GOBP_POSITIVE_REGULATION_OF_BMP_SIGNALING_PATHWAY
GOBP_ANATOMICAL_STRUCTURE_REGRESSION
GOBP_P38MAPK_CASCADE
GOBP_POSITIVE_REGULATION_OF_MYELOID_LEUKOCYTE_MEDIATED_IMMUNITY
GOBP_HISTONE_H3_ACETYLATION
GOBP_NEUTROPHIL_ACTIVATION_INVOLVED_IN_IMMUNE_RESPONSE
GOBP_REGULATION_OF_P38MAPK_CASCADE
GOBP_POSITIVE_REGULATION_OF_P38MAPK_CASCADE
GOBP_POSITIVE_REGULATION_OF_CHONDROCYTE_DIFFERENTIATION
GOBP_REGULATION_OF_PROTEIN_ACETYLATION
GOBP_HEAD_MORPHOGENESIS
GOBP_NEGATIVE_REGULATION_OF_EXOCYTOSIS
GOBP_NEGATIVE_REGULATION_OF_CD4_POSITIVE_ALPHA_BETA_T_CELL_ACTIVATION
GOBP_POSITIVE_REGULATION_OF_T_CELL_PROLIFERATION
GOBP_REGULATION_OF_BIOLOGICAL_PROCESS_INVOLVED_IN_SYMBIOTIC_INTERACTION
GOBP_NEGATIVE_REGULATION_OF_CD4_POSITIVE_ALPHA_BETA_T_CELL_DIFFERENTIATION
GOBP_NEGATIVE_REGULATION_OF_INTERLEUKIN_12_PRODUCTION
GOBP_HISTONE_H2A_ACETYLATION
GOBP_REGULATION_OF_T_HELPER_1_TYPE_IMMUNE_RESPONSE
GOBP_REGULATION_OF_RESPONSE_TO_TUMOR_CELL
GOBP_RESPONSE_TO_GROWTH_HORMONE
GOBP_ANTIMICROBIAL_HUMORAL_IMMUNE_RESPONSE_MEDIATED_BY_ANTIMICROBIAL_PEPTIDE
GOBP_NEGATIVE_REGULATION_OF_INTERLEUKIN_10_PRODUCTION
GOBP_CELLULAR_RESPONSE_TO_GROWTH_HORMONE_STIMULUS
GOBP_INTERLEUKIN_12_PRODUCTION
GOBP_POSITIVE_REGULATION_OF_ACTIVATED_T_CELL_PROLIFERATION
GOBP_INTERLEUKIN_10_PRODUCTION
GOBP_KILLING_OF_CELLS_OF_ANOTHER_ORGANISM
GOBP_T_HELPER_1_TYPE_IMMUNE_RESPONSE
GOBP_ACTIVATED_T_CELL_PROLIFERATION
GOBP_REGULATION_OF_DNA_STRAND_ELONGATION
GOBP_T_HELPER_1_CELL_DIFFERENTIATION
-2
-1
0
1
2
NES
pval
0.0125
0.0100
0.0075
0.0050
0.0025
0.0000

Supplementary Figure 2. Collagen-related gene ontology terms are enriched as regulators of collagen uptake in CRISPRi and CRISPRa screens. (a-d) The top twenty positive and negatively performing gene sets are indicated with associated statistics for each screen as indicated, among all 10,561 Gene Ontology gene sets. Collagen-related gene sets are highlighted with red boxes (i.e. those with 'Collagen' in the GO gene set name). NES = Normalized Enrichment Score. Statistics: GSEA algorithm (based on the Kolmogorov-Smirnov test), as described in the Methods section; unadjusted p-values are shown. Source data are provided in Supp. Data 1 and as a Source Data file.

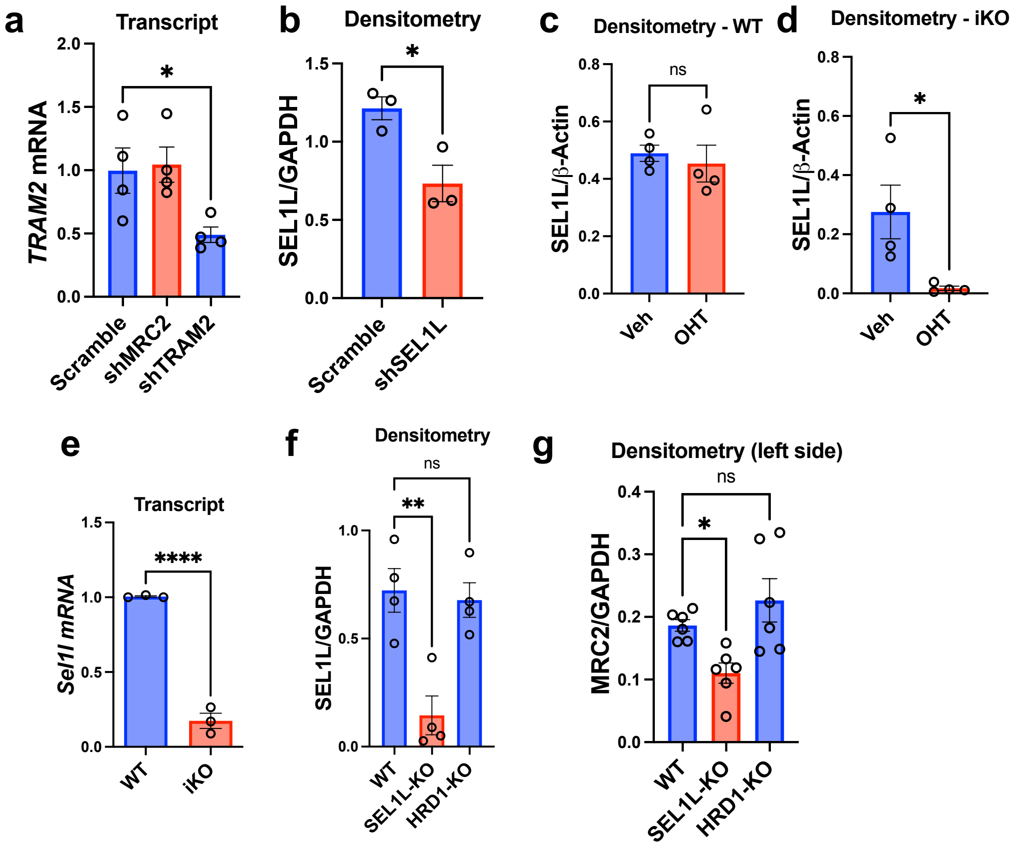
a
Transcript
TRAM2 mRNA
2.0
1.5
1.0
0.5
0.0
*
Scramble
shMRC2
shTRAM2
b
Densitometry
SEL1L/GAPDH
1.5
1.0
0.5
0.0
*
Scramble
shSEL1L
c
Densitometry - WT
SEL1L/β-Actin
0.8
0.6
0.4
0.2
0.0
ns
Veh
OHT
d
Densitometry - iKO
SEL1L/β-Actin
0.8
0.6
0.4
0.2
0.0
*
Veh
OHT
e
Transcript
Sel1l mRNA
1.5
1.0
0.5
0.0
****
WT
iKO
f
Densitometry
SEL1L/GAPDH
1.0
0.5
0.0
ns
**
WT
SEL1L-KO
HRD1-KO
g
Densitometry (left side)
MRC2/GAPDH
0.4
0.3
0.2
0.1
0.0
ns
*
WT
SEL1L-KO
HRD1-KO

Supplementary Figure 3. Validation of different tools used in this manuscript. (a) Q-RT-PCR in U937 cells after shRNA treatment as indicated vs. Scramble control. N=4 per group, p=0.0160. (b) Additional densitometry of data from Fig. 5c. N=4 per group, p=0.0253. (c-d) Additional densitometry of data from Fig. 5f. N=4 per group; p=0.6266 (c), p=0.0296 (d). (e) Q-RT-PCR in MEFs after OHT treatment for genes as indicated. N=3 per group, p<0.0001. (f) Additional densitometry of data from Fig. 6b. N=4 per group, p=0.0045 (WT vs. SEL1L-KO), p>0.9999 (WT vs. HRD1-KO). (g) Densitometry of the left side of Fig. 6e. N=6 per group, p=0.0427 (WT vs. SEL1L-KO), p=0.3644 (WT vs. HRD1-KO). Data are shown as the mean ± SEM. Statistics: (a) one-way ANOVA with post-hoc Bonferroni testing; (b-e) unpaired Student's *t*-test (two-sided); (f, g) repeated-measures ANOVA with post-hoc Bonferroni testing. * p < 0.05, ** p < 0.01, **** p < 0.0001, ns = not significant. Source data are provided as a Source Data file.

a

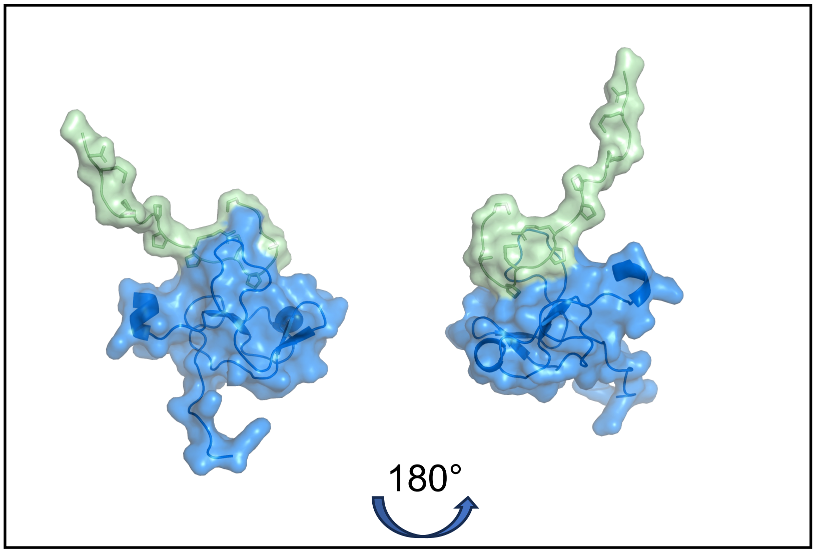

Green: collagen peptide; Blue: FN2 domain of SEL1L

b

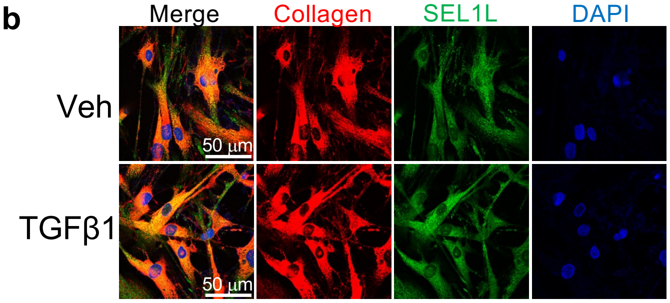

Supplementary Figure 4. Additional support of SEL1L and collagen interaction. (a) Predicted docking of collagen peptide with FN2 domain of SEL1L. (b) Representative confocal immunofluorescence images of MRC5 fibroblasts treated as indicated or vehicle control.

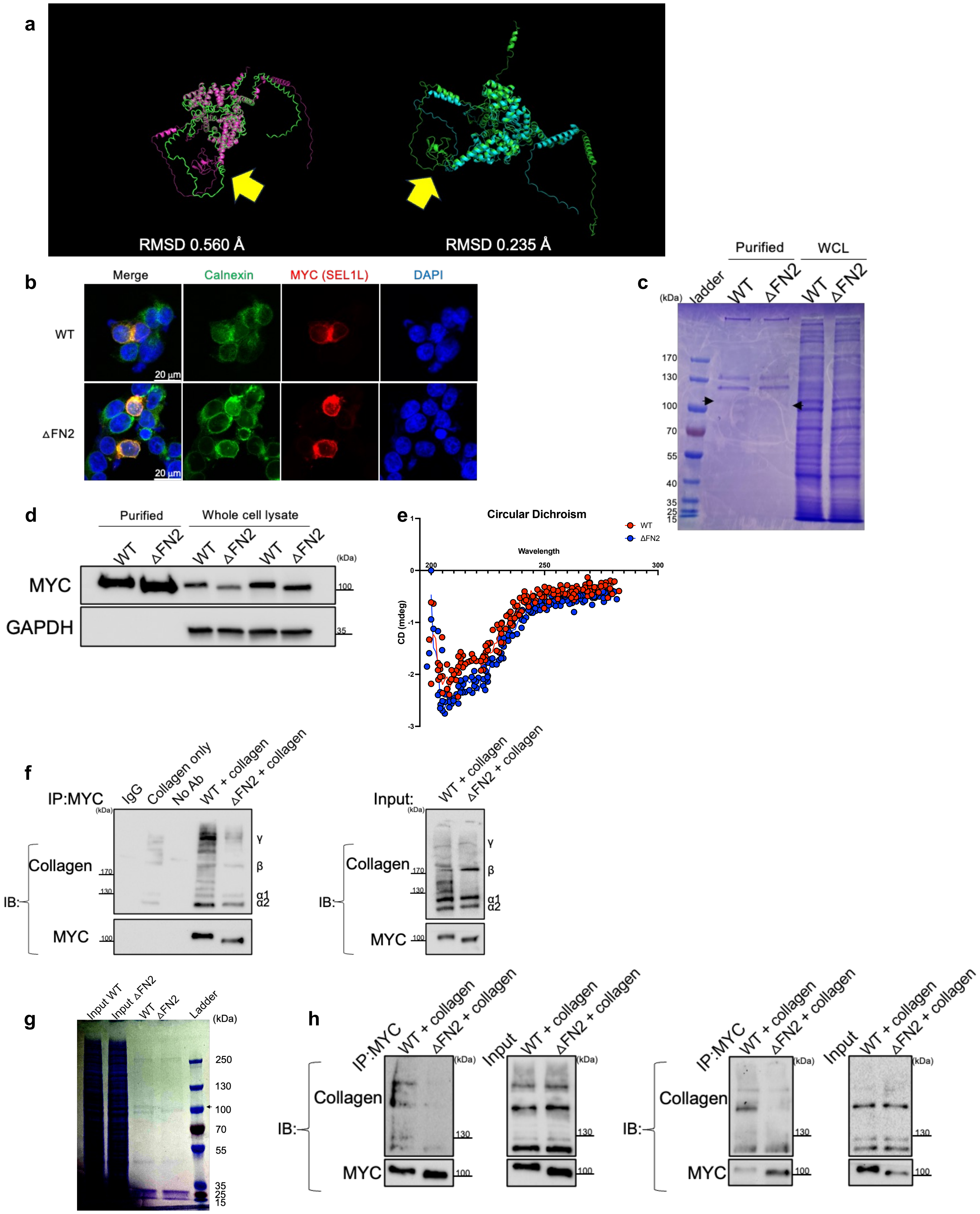

a
RMSD 0.560 Å
RMSD 0.235 Å
b
Merge
Calnexin
MYC (SEL1L)
DAPI
WT
ΔFN2
20 μm
c
Purified
WCL
Ladder
WT
ΔFN2
(kDa)
170
130
100
70
55
40
35
25
15
d
Purified
Whole cell lysate
MYC
GAPDH
(kDa)
e
Circular Dichroism
WT
ΔFN2
Wavelength
CD (mdeg)
f
IP:MYC
IgG
Collagen only
No Ab
WT + collagen
ΔFN2 + collagen
Collagen
MYC
IB:
γ
β
α1
α2
Input:
g
Input WT
Input ΔFN2
Ladder
250
h
Input

Supplementary Figure 5. FN2 domain deletion mutant interferes with collagen binding but is otherwise stable. (a) Alignment of predicted structures of full-length SEL1L and ∆FN2 SEL1L. Two different views and alignments are shown. Root-mean squared distance (RMSD) is displayed. Yellow arrow indicates the FN2 domain (in the pink structure on the left; in the green structure on the right). (b) Representative confocal immunofluorescence images of HEK293T cells transfected with MYC-tagged WT or ∆FN2 SEL1L constructs (at low transfection efficiency), co-stained with ER marker Calnexin. (c) Representative Coomassie Brilliant Blue staining of Whole Cell Lysate (WCL) or purified WT or ∆FN2 SEL1L proteins, representative of N=4 independent experiments; bands excised for proteomics indicated with arrowheads. (d) Western blot of purified WT or ∆FN2 SEL1L proteins compared with input cell lysates, representative of N=3 independent experiments. (e) Circular Dichroism spectra of purified WT or ∆FN2 proteins. (f) Western blot of co-incubated purified either WT or ∆FN2 SEL1L proteins with rat tail collagen, after immunoprecipitation with anti-MYC antibody (with input and additional controls as indicated), representative of N=3 independent experiments. 'Collagen only' indicates incubation of rat tail collagen with IP beads without co-incubation with purified MYC-tagged protein. (g) Representative Coomassie Brilliant Blue staining of purified WT or ∆FN2 SEL1L using anti-FLAG method, representative of N=4 independent experiments; arrowhead indicates expected MW of SEL1L. (h) Western blots of either WT or ∆FN2 SEL1L proteins purified via anti-FLAG method co-incubated with rat tail collagen, after co-immunoprecipitation with anti-MYC antibody (with input controls as indicated); two experiments shown (left and right), representative of N=3 independent experiments. Source data are provided as a Source Data file.
