# Supplementary material for: Genome-wide screens identify SEL1L as an intracellular rheostat controlling collagen turnover
Source: Nat Commun. 2024 Feb 20;15:1531. doi: 10.1038/s41467-024-45817-8 (PMC10879544; doi:10.1038/s41467-024-45817-8)
Supplement: Supplementary file 3 — Description of Additional Supplementary Files [file 41467_2024_45817_MOESM3_ESM.pdf]

Description of Additional Supplementary Files

Supplementary Data Legends

Supplementary Data 1: Gene-level data from CRISPRi and CRISPRa screens of collagen uptake as analyzed via MAGeCK. Statistics: Robust-rank aggregation scores computed with MAGeCK as described in the Methods, with both unadjusted p-values and significance values adjusted for multiple comparisons shown.

Supplementary Data 2: Primer, shRNA and gRNA sequences used in this manuscript.

Supplementary Data 3: Proteomic identification of excised gel bands.

Supplementary Data 4: Demographic and clinical data on the sources of human lungs used in this manuscript.
