# Supplementary material for: Genome-wide screens identify SEL1L as an intracellular rheostat controlling collagen turnover
Source: Nat Commun. 2024 Feb 20;15:1531. doi: 10.1038/s41467-024-45817-8 (PMC10879544; doi:10.1038/s41467-024-45817-8)
Supplement: Supplementary file 8 — Reporting Summary [file 41467_2024_45817_MOESM8_ESM.pdf]

Corresponding author(s): Kamran Atabai, Michael Podolsky

Last updated by author(s): Jan 10, 2024

Reporting Summary

Nature Portfolio wishes to improve the reproducibility of the work that we publish. This form provides structure for consistency and transparency in reporting. For further information on Nature Portfolio policies, see our Editorial Policies and the Editorial Policy Checklist.

Statistics

For all statistical analyses, confirm that the following items are present in the figure legend, table legend, main text, or Methods section.

n/a	Confirmed	
☐	☒	The exact sample size (*n*) for each experimental group/condition, given as a discrete number and unit of measurement
☐	☒	A statement on whether measurements were taken from distinct samples or whether the same sample was measured repeatedly
☐	☒	The statistical test(s) used AND whether they are one- or two-sided<br>*Only common tests should be described solely by name; describe more complex techniques in the Methods section.*
☐	☒	A description of all covariates tested
☐	☒	A description of any assumptions or corrections, such as tests of normality and adjustment for multiple comparisons
☐	☒	A full description of the statistical parameters including central tendency (e.g. means) or other basic estimates (e.g. regression coefficient) AND variation (e.g. standard deviation) or associated estimates of uncertainty (e.g. confidence intervals)
☐	☒	For null hypothesis testing, the test statistic (e.g. *F*, *t*, *r*) with confidence intervals, effect sizes, degrees of freedom and *P* value noted<br>*Give P values as exact values whenever suitable.*
☒	☐	For Bayesian analysis, information on the choice of priors and Markov chain Monte Carlo settings
☒	☐	For hierarchical and complex designs, identification of the appropriate level for tests and full reporting of outcomes
☐	☒	Estimates of effect sizes (e.g. Cohen's *d*, Pearson's *r*), indicating how they were calculated

Our web collection on statistics for biologists contains articles on many of the points above.

Software and code

Policy information about availability of computer code

Data collection	FACSDiva 6 or higher; Accuri c6 software v1.0; Bio-Rad CFX software 2.0; Applied Biosystems QuantStudio 5; Leica LASX 4.4; Bio-Rad XRS+ Image Lab 5.0; Synergy H1 Plate Reader Gen5 3.02; Nikon NIS-Elements 5.20; Chirascan V100
Data analysis	Python v3 scripts referenced in text (ScreenProcessing: https://github.com/mhorlbeck/ScreenProcessing); MAGeCK 0.5.9; R 4.1.1 (ggplot2 package); GSEA 4.2.3; GEO2R (R 4.2.2, Biobase 2.58.0, GEOquery 2.66.0, limma 3.54.0); FlowJo 7.6.1; Fiji 1.53c; Graphpad Prism 9; PyMol 2.3; MaxQuant v2

For manuscripts utilizing custom algorithms or software that are central to the research but not yet described in published literature, software must be made available to editors and reviewers. We strongly encourage code deposition in a community repository (e.g. GitHub). See the Nature Portfolio guidelines for submitting code & software for further information.

Data

Policy information about availability of data

All manuscripts must include a data availability statement. This statement should provide the following information, where applicable:

- Accession codes, unique identifiers, or web links for publicly available datasets
- A description of any restrictions on data availability
- For clinical datasets or third party data, please ensure that the statement adheres to our policy

Source data are provided with this paper. All data generated or analyzed during this study are included in this published article (and its supplementary information

files). Prior data from the Gene Expression Omnibus (GEO) used for re-analysis can be found on the GEO database: GSE136831 [https://www.ncbi.nlm.nih.gov/geo/query/acc.cgi?acc=GSE136831], GSE135893 [https://www.ncbi.nlm.nih.gov/geo/query/acc.cgi?acc=GSE135893], GSE121611 [https://www.ncbi.nlm.nih.gov/geo/query/acc.cgi?acc=GSE121611], GSE128033 [https://www.ncbi.nlm.nih.gov/geo/query/acc.cgi?acc=GSE128033], GSE132771 [https://www.ncbi.nlm.nih.gov/geo/query/acc.cgi?acc=GSE132771], GSE110147 [https://www.ncbi.nlm.nih.gov/geo/query/acc.cgi?acc=GSE110147], GSE70867 [https://www.ncbi.nlm.nih.gov/geo/query/acc.cgi?acc=GSE70867], GSE37858 [https://www.ncbi.nlm.nih.gov/geo/query/acc.cgi?acc=GSE37858], GSE40151 [https://www.ncbi.nlm.nih.gov/geo/query/acc.cgi?acc=GSE40151].

Research involving human participants, their data, or biological material

Policy information about studies with human participants or human data. See also policy information about sex, gender (identity/presentation), and sexual orientation and race, ethnicity and racism.

Reporting on sex and gender	Data on sex are reported in supplementary data 4.
Reporting on race, ethnicity, or other socially relevant groupings	Data on race/ethnicity are reported in supplementary data 4.
Population characteristics	The normal specimens represent a breadth of ages (most within ages 20 - 80) with equal numbers of male and female sources. The proportion of men is higher for IPF specimens (approximately 60:40, M:F), and ages > 50, consistent with disease epidemiology.
Recruitment	Normal human lung tissue from deceased donors of different ages was obtained from lungs not used by the Northern California Transplant Donor Network according to an IRB-exempted protocol led by Dr. Wolters; IPF lung specimens were obtained from explanted lungs removed during lung transplantation, in an IRB-approved protocol led by Dr. Wolters.
Ethics oversight	UCSF IRB approved Dr. Wolters' protocol; WCMC IRB determined Dr. Podolsky's protocol as non-human subjects research

Note that full information on the approval of the study protocol must also be provided in the manuscript.

Field-specific reporting

Please select the one below that is the best fit for your research. If you are not sure, read the appropriate sections before making your selection.

☒ Life sciences ☐ Behavioural & social sciences ☐ Ecological, evolutionary & environmental sciences

For a reference copy of the document with all sections, see nature.com/documents/nr-reporting-summary-flat.pdf

Life sciences study design

All studies must disclose on these points even when the disclosure is negative.

Sample size	Sample size for in vitro cell culture experiments was generally N=4-8 biological replicates, based on a power calculation with the following characteristics: need to detect a 50% difference in the readouts assuming standard error of 10-20%, with a power of 90% and an alpha of 0.05, all based on our prior work in similar types of experiments. Coverage for CRISPR screening and rationale (as related to sgRNA library size) are described in the text of the manuscript.
Data exclusions	Data were not excluded.
Replication	Replication was performed as described in the methods section, using independent biological replicates. All attempts at replication in which internal technical controls performed as expected were successful.
Randomization	Allocation of cells into groups for intervention was random.
Blinding	Blinding of investigators to group allocation for cell experiments was done to the extent feasible.

Reporting for specific materials, systems and methods

We require information from authors about some types of materials, experimental systems and methods used in many studies. Here, indicate whether each material, system or method listed is relevant to your study. If you are not sure if a list item applies to your research, read the appropriate section before selecting a response.

Materials & experimental systems

n/a	Involved in the study
	☒ Antibodies
	☒ Eukaryotic cell lines
☒	Palaeontology and archaeology
☒	Animals and other organisms
☒	Clinical data
☒	Dual use research of concern
☒	Plants

Methods

n/a	Involved in the study
☒	ChIP-seq
	☒ Flow cytometry
☒	MRI-based neuroimaging

Antibodies

Antibodies used

MRC2 (anti-mouse: AF4789; anti-human: AF5770; R&D Systems, Bio-Techne) , SEL1L (Novus Biologicals: NBP2-93746; ABCAM: ab78298), collagen I (Southern Biotech: 1310-01), human procollagen I alpha 1 (Novus Biologicals: AF6220), MYC (Cell Signaling Technology, 9B11) GAPDH (Cell Signaling Technology, 14C10), beta-Actin (Cell Signaling Technology, 13E5) , OS9 (Novus Biologicals: NB100-519), CD147 (Proteintech, 11989-1-AP), SHH (Cell Signaling Technology, C9C5), IRE1a (Cell Signaling Technology, 14C10), Vinculin (Cell Signaling Technology, E1E9V), horseradish peroxidase–conjugated anti-Rabbit (Cell Signaling Technology, 7074), horseradish peroxidase–conjugated anti-Sheep (R&D Systems, Bio-Techne, HAF016), horseradish peroxidase–conjugated anti-Goat (Santa Cruz Biotechnology, sc-2354), Alexa 647-conjugated donkey anti-sheep (Invitrogen, A-21448), Calnexin (Cell Signaling Technology, C5C9), MYC (Santa Cruz Biotechnology, 9E10), SEL1L (for Immunofluorescence - gift from Dr. Qi), Type I collagen (Rockland, 600-401-103), anti-Rabbit or Goat or Sheep or anti-mouse conjugated to Alexa Fluor 488 or 594 or 647 (Invitrogen, A-1105, A-21207, A-21448, A-31571 or A-11015)

Validation

MRC2 (IF, Western, flow; anti-human and mouse), SEL1L (IF and Western, anti-human and mouse), MYC (IF and Western, anti-human and mouse): validated with overexpression and/or KO cell lines
OS9 (Western, anti-human), CD147 (Western, anti-human), SHH (Western, anti-human), IRE1a (Western, anti-human): validated based on expected MW and effect from interfering with ERAD
Calnexin (IF, anti-human): validated based on expected distribution of staining; manufacturer validated with antigen-specific vs. control peptides, and notes >25 relevant citations validating the antibody)
Type I Collagen (SB, Western and IF, human and mouse): validated with overexpression cell lines
Type I Collagen (Rockland, IF, anti-human): validated with overexpression cell lines
Procollagen I alpha 1 (Western, anti-human): validated with overexpression cell lines
Vinculin (Western, anti-human), GAPDH (Western, anti-human and mouse), beta-Actin (Western, anti-human and mouse): verified based on MW of expected band at correct size, and prior usage in our laboratory, manufacturer statements based on recognition of the specific endogenous antigens

Eukaryotic cell lines

Policy information about cell lines and Sex and Gender in Research

Cell line source(s)

U937, MRC5, HEK293T: ATCC
modified MEF, modified HEK293T: Dr. Qi's laboratory (as described in manuscript)

Authentication

Commercial cell lines U937, MRC5 and HEK293T were purchased fresh from ATCC but were not further authenticated; modified cell lines from Dr. Qi's laboratory were validated to have expected gene KO by QPCR and Western blotting.

Mycoplasma contamination

Cell lines were not recently tested for Mycoplasma contamination

Commonly misidentified lines
(See ICLAC register)

No commonly misidentified cell lines were used in the study.

Plants

Seed stocks

n/a

Novel plant genotypes

n/a

Authentication

n/a

Flow Cytometry

Plots

Confirm that:

☒ The axis labels state the marker and fluorochrome used (e.g. CD4-FITC).

☒ The axis scales are clearly visible. Include numbers along axes only for bottom left plot of group (a 'group' is an analysis of identical markers).

☒ All plots are contour plots with outliers or pseudocolor plots.

☒ A numerical value for number of cells or percentage (with statistics) is provided.

Methodology

Sample preparation	Single cell suspension was made in 2%BSA/PBS or 5%FCS/PBS as described in the text.
Instrument	FACSVerse, FACSAriaII, or Accuri c6
Software	FACSDiva or Accuri c6 software were used for acquisition; FlowJo was used for analysis.
Cell population abundance	For any sorting experiments, cells were manually counted afterwards to determine cell numbers obtained; purity of post-sort populations, for example when making clonal populations of dCas9-KRAB or dCas9-VP64 expressing cells, was determined via flow cytometric analysis after any initial sorting.
Gating strategy	FSC/SSC gates were initially made to include only live cells (to gate out debris, cell clumps, or dead cells), followed by gating for singlets (generally based on FSC-A vs. FSC-H), followed by analysis of fluorescent collagen uptake or cell surface marker staining as described in the text. Only single color flow cytometric analysis was performed in this manuscript.

☐ Tick this box to confirm that a figure exemplifying the gating strategy is provided in the Supplementary Information.
